# A Review: Subcritical Water Extraction of Organic Pollutants from Environmental Matrices

**DOI:** 10.3390/molecules29010258

**Published:** 2024-01-03

**Authors:** Erdal Yabalak, Mohammad Tahir Aminzai, Ahmet Murat Gizir, Yu Yang

**Affiliations:** 1 Department of Nanotechnology and Advanced Materials, Mersin University, TR-33343 Mersin, Türkiye; 2 Department of Organic Chemistry, Faculty of Chemistry, Kabul University, Kabul 1006, Afghanistan; mt.aminzai@gmail.com; 3 Department of Chemistry, Faculty of Science, Mersin University, TR-33343 Mersin, Türkiye; agizir@mersin.edu.tr; 4 Department of Chemistry, East Carolina University, Greenville, NC 27858, USA

**Keywords:** environmental matrices, organic pollutants, pesticides, polycyclic aromatic hydrocarbons, polychlorinated biphenyls, subcritical water extraction

## Abstract

Most organic pollutants are serious environmental concerns globally due to their resistance to biological, chemical, and photolytic degradation. The vast array of uses of organic compounds in daily life causes a massive annual release of these substances into the air, water, and soil. Typical examples of these substances include pesticides, polychlorinated biphenyls (PCBs), and polycyclic aromatic hydrocarbons (PAHs). Since they are persistent and hazardous in the environment, as well as bio-accumulative, sensitive and efficient extraction and detection techniques are required to estimate the level of pollution and assess the ecological consequences. A wide variety of extraction methods, including pressurized liquid extraction, microwave-assisted extraction, supercritical fluid extraction, and subcritical water extraction, have been recently used for the extraction of organic pollutants from the environment. However, subcritical water has proven to be the most effective approach for the extraction of a wide range of organic pollutants from the environment. In this review article, we provide a brief overview of the subcritical water extraction technique and its application to the extraction of PAHs, PCBs, pesticides, pharmaceuticals, and others form environmental matrices. Furthermore, we briefly discuss the influence of key extraction parameters, such as extraction time, pressure, and temperature, on extraction efficiency and recovery.

## 1. Introduction

An enormous number of organic chemicals are used daily to meet human physiological needs and maintain a healthy and better quality of life. As a result, large quantities of organic pollutants (OPs), including insecticides, herbicides, fungicides, polychlorinated biphenyls (PCBs), and polycyclic aromatic hydrocarbons (PAHs), are released into the environment [1,2,3]. The majority of these OPs are resistant to biodegradation, persist for a long time in the environment, and migrate from one place to another; for these reasons, they are called persistent organic pollutants (POPs) [4]. In contrast to the high solubility of OPs in non-polar solvents and edible oils, they exhibit relatively low solubility in water. Moreover, the addition of extra rings to the PAHs decreases their solubility. These chemicals bio-accumulate in the food chain and are widespread toxic contaminants that endanger humans, animals, and ecosystems [5]. Hence, POPs are found in small amounts in the human body and are more prevalent in foods high in fat, such as meat, fish, eggs, and milk, due to their lipophilic nature [6]. Exposure to these contaminants causes a variety of health issues, including cancer, diabetes, heart problems, endocrine disorders, and reproductive system problems [7]. As a consequence, effective methods for OP extraction and detection are essential for determining the degree of contamination and assessing the potential risks it causes to the ecosystem.

**Figure 1:**
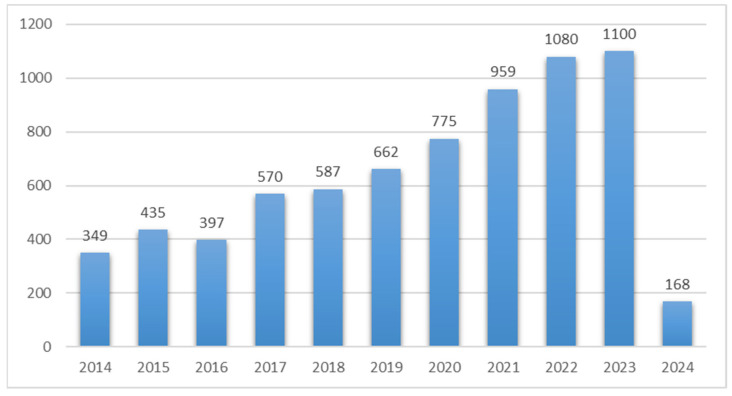
Year-wise publication of subcritical water extraction papers between 2014 and 2024; data were retrieved from the ScienceDirect database.

Liquid–liquid extraction (LLE) and solid-phase extraction (SPE) are two of the most well-known and frequently used methods for the extraction of OPs from different environmental samples [8]. However, these classical extraction techniques frequently have several drawbacks, including being difficult to automate, complicated and time-consuming, and requiring large quantities of glassware and organic solvents, which are frequently hazardous to the environment [8,9]. As an alternative, several new green extraction and separation technologies, such as pressurized liquid extraction (PFE) [10], microwave-assisted extraction (MAE) [11], ultrasonic-assisted extraction (UAE) [12], supercritical fluid extraction (SFE) [13,14], and subcritical water extraction (SBWE) [14,15], have recently been developed to reduce extraction times and organic solvent requirements for OPs extraction from liquid and solid matrices. However, SBWE has been utilized to extract a range of OPs from environmental matrices and is the most promising of the group. The subcritical water extraction technique has been the focus of much study for the past ten years (Figure 1). It has been used in the pharmaceutical industry to extract natural products and essential oils, as well as in the environmental sector to extract contaminants.

SBWE is a relatively new technique for the extraction of organic compounds using pure liquid water under critical temperature and pressure (Tc = 374.15 °C, Pc = 22.1 MPa) [16]. The primary benefits of SBWE are attributed to the usage of water instead of toxic organic solvents and the reduced extraction time, which considerably reduces the cost of extraction processes, as well as environmental pollution [17]. The solvent’s fundamental characteristic that identifies its polarization is its dielectric constant (ε). For instance, water has a dielectric constant of about 80 in mild extraction conditions (25 °C, 0.1 MPa), which drops to 25 when the temperature and pressure are increased to 250 °C and 2.5 Mpa, respectively [18]. As a result, water can behave identically to acetonitrile, methanol, and ethanol due to the tunable polarity of subcritical water, which allows for the extraction of a variety of polar or less polar organic compounds by adjusting extraction conditions (pressure and temperature) [19,20,21].

**Figure 2:**
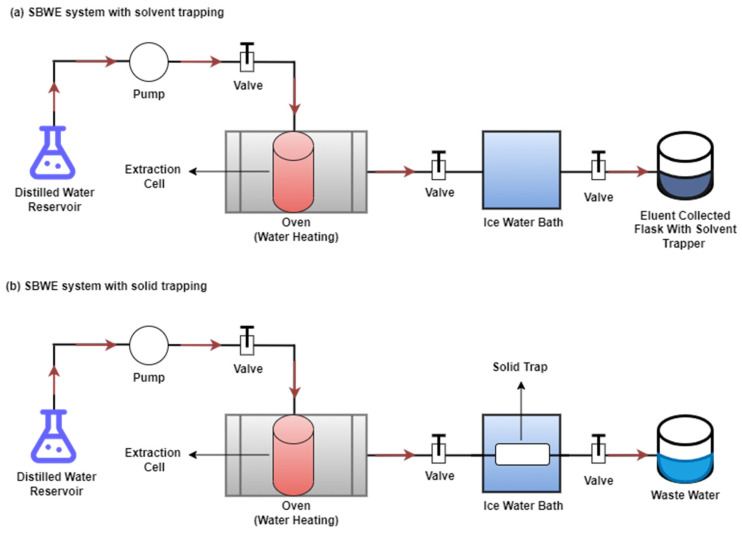
SBWE system with (**a**) solvent trapping and (**b**) solid trapping.

The SBWE process involves two types of extraction modes: static extraction (discrete mode) and dynamic extraction (continuous flow mode) [16,22]. These extraction modes can be used individually or together. However, due to the allowance for the continuous flow of fresh water through the extraction vessel, the compounds are extracted continuously, and thus the recovery efficiency of the dynamic mode is much higher than that of the static mode. In order to collect extracted analytes, SBWE systems can utilize both solvent and solid trapping, as shown in Figure 2 [23,24]. However, sorbent-type trapping has been used more commonly due to the limitations of solvent trapping.

As shown in Figure 2, distilled water is pumped from the water reservoir to the extraction cell using a syringe pump and heated in an oven using pre-heating coils. The eluent, which contains the extracted organic compounds, is cooled by being passed through an ice-water bath and then collected in a flask containing an organic solvent trapper (Figure 2a). While utilizing a solid trapper, after the extraction step, the extracted organic compounds are cooled and collected on sorbent at the same time (Figure 2b).

In addition to extracting diverse bioactive substances (such as polyphenols, pigments, essential oils, flavonoids, and peptides) from various raw materials, SBWE is also capable of extracting and remediating a wide range of contaminants (such as pesticides, PAHs, and PCBs) from various environmental matrices [25,26,27,28,29,30]. In this study, we present a comprehensive and systematic explanation of the SBWE approach for extracting organic pollutants from environmental matrices, including pesticides, pharmaceuticals, PAHs, PCBs, pharmaceuticals, and phthalates. Additionally, the comparison of SBWE with other analytical methods and the impact of extraction temperature, pressure, and time on recovery and extraction efficiencies of organic pollutants are discussed briefly,

## 2. Types of Analytes Extracted

This review article provides a comprehensive overview of the application of subcritical water in the extraction of various organic contaminants (PAHs, PCBs, insecticides, herbicides, fungicides, pharmaceuticals, phthalates, and others) from a variety of solid and liquid environmental matrices (waters, soils, dust, sludges, sediments, and others). 

### 2.1. PAHs

**Figure 3:**
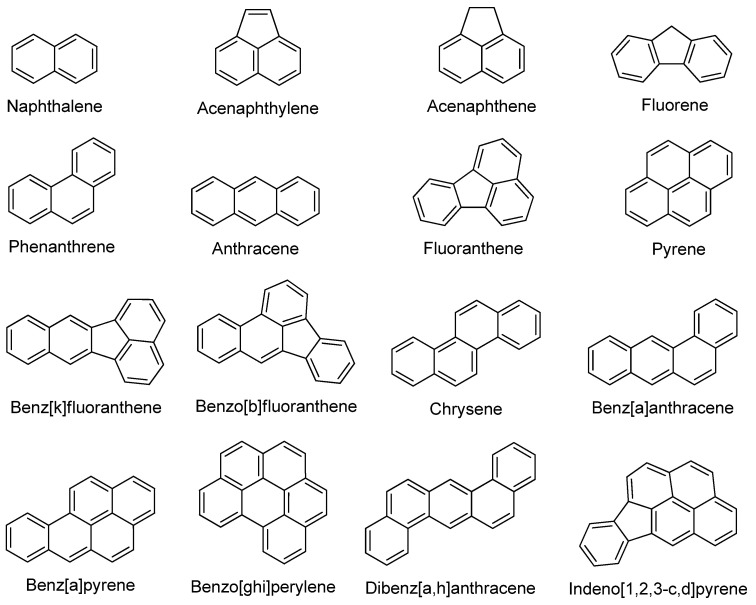
The chemical structures of the 16 typical PAHs identified by the US-EPA as the most hazardous and carcinogenic.

PAHs refer to a class of organic compounds consisting of two or more fused benzene rings in the molecule [31]. Based on fused benzene rings, PAHs can be classified as light PAHs (2–4 rings) or heavy PAHs (4–6 rings) [32]. PAHs are widespread chemical contaminants that are frequently found in the air, soil, and water [33]. They are largely produced by the incomplete combustion of organic substances (e.g., oil, coal, gasoline, and wood) and are typically toxic, mutagenic, and carcinogenic [34,35]. Figure 3 represents some of the most hazardous and carcinogenic PAHs as specified by the United States Environmental Protection Agency (US-EPA).

The adverse impacts of PAHs on human health are mostly determined by human exposure to a precise quantity of PAHs, the duration of exposure, the mode of exposure, and the inherent toxicity of PAHs [36,37]. Short-term impacts of PAHs on human health include symptoms such as anxiety, vomiting, nausea, and eye irritation. Reduced immunological function, vision problems, kidney and liver damage, respiratory issues, signs of asthma, alterations to lung function, and skin redness and inflammation are the chronic health effects of long-term human exposure to PAHs [35,37,38,39]. In fact, cancer is the most serious and significant outcome of PAH intoxication [40]. PAHs are highly soluble in nonpolar solvents and edible oils but have limited solubility in water [41]. Regardless of the hydrophobic properties of PAHs, a broad range of extraction techniques, such as dispersive solid-phase extraction (d-SPE) [42], magnetic solid-phase extraction (MSPE) [43], stir bar sorptive extraction (SBSE) [44], fabric phase sorptive extraction (FPSE) [45], SBWE [46], and SFE [47], have been developed. The application of SFE for the extraction of PAHs from the environment has become more apparent; however, supercritical water requires extremely high temperatures and pressures (T > 374 °C and P > 221 bar), and it is extremely corrosive [48,49]. SBWE, on the other hand, is an emerging technology that utilizes superheated water as a solvent instead of toxic organic solvents [50]. Several studies have investigated clean-up of PAHs and demonstrated the capability of extracting PAHs from diverse environmental matrices using subcritical water [46,51]. The extraction of PAHs from environmental matrices utilizing subcritical water as a green solvent is illustrated in Table 1 [14].

Hawthorne et al. obtained quantitative extractions of PAHs ranging from 86 to 100% in just 15 min under very mild conditions (e.g., 250 °C and 50 bar) [14]. Solid-phase micro extraction (SPME) has been shown to be an efficient method for quantitatively extracting organic molecules from water. Thus, Hageman reported quantitative PAH determination from soil using the SBWE/SPME method at 250 °C [52]. Subcritical water could be effectively utilized for selective extraction of polar, moderately polar, and nonpolar organic compounds by merely adjusting water temperatures (50–300 °C) and pressures (5–100 atm). Thus, at 250 or 300 °C and pressures between 50 and 100 atm, Yang et al. observed selective extraction of PAHs over phenols, BTEX, and n-alkanes from petroleum waste sludges, soil, and a spent catalyst. PAH remediation using subcritical water at a pilot scale in contaminated soil (8 kg of soil sample) was performed by Lagadec et al., and all PAHs were reduced to undetectable levels (0.5 ppm) in as short as 35 min at 275 °C and 100 bar [48]. Hawthorne et al. demonstrated that the extraction and recovery of PAHs from polluted soils and sediments are made very simple and quick by coupling subcritical water extraction with SPE sorbent disc collection [51].

More than 90% of the 15 typical PAHs were quantitatively extracted and collected on a sorbent disk at an extraction temperature of 250 °C for 60 min. According to Fernandez-Perez et al., the use of sodium dodecyl sulfate (SDS) at 50 bar and 150 °C, for 10 min of dynamic extraction and 15 min of static extraction is an effective way to improve the extraction of non-polar organics from soils [53]. Very high recovery percentages were achieved, ranging from 73.6 to 110.4 using SD-water, compared to 30–80% using only water as an extracting agent. Mcgowin et al. used static SBWE and SPE to assess PAHs in certified reference sediment (CRM 104) and found that an extraction length of 20 min and a temperature of 150 °C offered the highest extraction recovery [54]. Additionally, they demonstrated that using C-18 resin as an alternate sorbent significantly increased the percentage of PAHs recovered. According to Moreno et al., it was feasible to quantitatively extract PAHs at concentrations as high as 8522 mg kg^−1^ under SWBE conditions, and extraction recoveries for PAHs from Mexican hydrocarbon-contaminated soil varied from 95 to 100% with an average uncertainty of ±1.2% [60]. Wang et al. discovered that subcritical water extraction combined with dispersive liquid–liquid microextraction (DLLME) produced a promising recovery rate ranging from 57.63% to 91.07% for the detection of monohydroxylated PAHs in sediment samples [61].

As a result, low molecular weight PAHs, such as naphthalene, are more water-soluble than other PAHs, allowing them to be extracted at lower temperatures (150–200 °C), whereas higher molecular weight PAHs require very high temperatures (200–300 °C) to achieve the best extraction results [46,62].

### 2.2. PCBs

**Figure 4:**
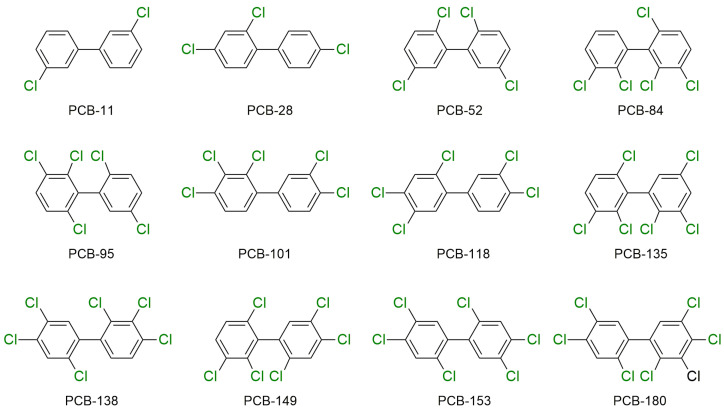
Most abundant PCBs in human maternal serum.

PCBs are a group of synthetic persistent organic pollutants that were once largely utilized as hydraulic fluids, heat-transfer fluids, lubricating fluids, plasticizers, and insulating fluids in some electrical equipment, such as capacitors and transformers [63]. These substances are among the most persistent xenobiotic pollutants, and can survive in different environmental situations for an extended period due to their significant chemical stability and minimal reactivity [64]. Humans are mainly exposed to PCBs by oral intake, inhalation, and skin adsorption [65]. PCBs have several negative consequences on human health, including cancer, skin and liver damage, birth defects, metabolic disorders, cardiovascular problems, immune system failure, and other health problems [66,67]. Thus, due to PCBs’ health risks, they are currently forbidden, and manufacturers stopped producing PCBs commercially in 1977. Products that were produced before the ban and that are discarded as garbage continue to gradually and continuously leak PCBs into the environment (soils, sewage, surface sludge, sediments, and surface water) [68,69,70,71]. The chemical structures of the 12 PCBs that are most abundant in human maternal serum are shown in Figure 4 [72].

Therefore, it has been crucial to accurately identify and eliminate PCBs from the ecosystem to minimize their harmful and carcinogenic health impacts. Comparable to how PAHs are extracted, PCBs can be quantitatively extracted using SBWE at 250 °C and 300 °C. Since PCBs are nonpolar molecules and only minimally soluble in water, the temperature of the water has a big impact on how efficiently PCBs can be extracted. Table 2 shows the extraction of PCBs from environmental matrices using SBWE.

At 250 °C and 50 atm, Yang et al. demonstrated effective removal (>99%) of PCBs from sediment in just 15 min [73], although in comparable reaction conditions, the extraction efficiency of the majority of PCBs from industrial soils was only >91% and 71–88% for highly chlorinated biphenyls. As a result, the extraction of the majority of PCBs, including highly chlorinated biphenyls (hexa- and hepta-chlorobiphenyls), was carried out at 300 °C (steam) and 50 atm, where it took less than 5 min to complete. Hartonen et al. reported a recovery rate of more than 85% for spiked PCBs from sea sand using a solid-phase trap packed with Tenax [74]. Then, Pross et al. investigated three extraction fluids (CO_2_, H_2_O, and SF_6_) for the extraction of spiking PCBs from soil [77]. They demonstrated that, of all studied fluids, water was the most effective in extracting PCBs. They also showed that using octadecylsilane (ODS) instead of Tenax might increase solid phase trapping efficiency, as the extraction recovery efficiency can reach over 95%. SWBE can be coupled to SPME for rapid estimation of PCB concentrations in soils and sediments or HPLC for more accurate and sensitive PCB analysis [78,79]. Li et al. devised an identical on-line SBWE-HPLC coupling system using a sorbent trap as an interface for extraction and analysis of chlorophenols, chloro- and methylanilines, caffeine, nitrotoluenes, and PCBs from the sand [75]. They also indicated that at 250 °C, all PCBs examined were efficiently extracted from sand, with recoveries of 87% or higher. Recently, at an extraction temperature of 225–250 °C, Islam et al. reported the total elimination of over 99% of PCBs from field-contaminated soil in 60 min [76].

### 2.3. Pesticides

**Figure 5:**
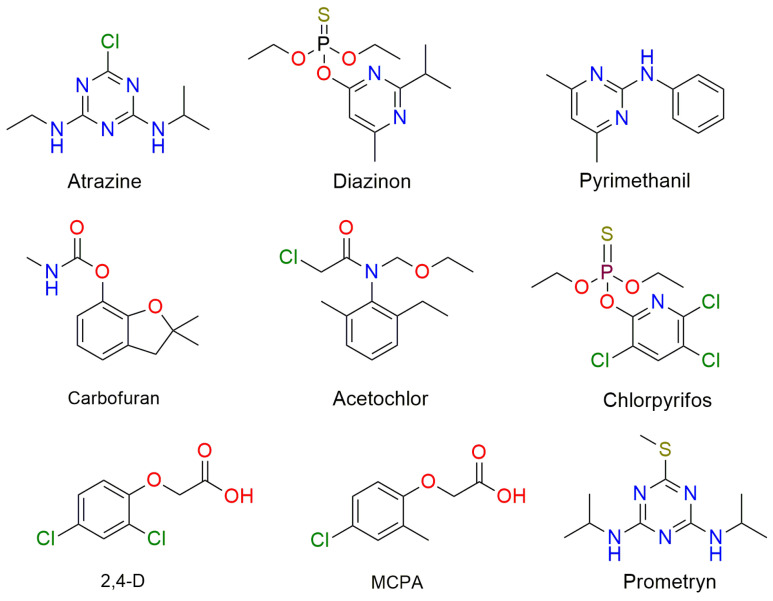
Chemical structure of some of the most abundant pesticides in surface water.

The term “pesticides” refers to a class of chemicals that are used to both protect crops from pests (such as insects, rodents, fungi, and weeds) and boost crop production. Hence, pesticides can be categorized as insecticides, rodenticides, fungicides, herbicides, and many more, depending on the species they are meant to kill [80,81,82]. China, the United States of America, Argentina, India, Japan, Canada, Brazil, France, Italy, and Thailand are the top pesticide consumers, using an average of 2 million tons of pesticides annually [83,84]. Among all pesticides consumed in the world, 80% are insecticides, 1.46% are fungicides, 15% are herbicides, and the remaining are other types of pesticides [85]. The excessive and continuous utilization of pesticides can eventually cause harm to non-target species, including humans, animals, plants, and several other beneficial organisms [86]. These persistent organic pollutants are frequently identified in soils because they are directly applied to them; one of the most comprehensive and recent examinations showed that 83% of the 317 agricultural soils studied contained at least one pesticide residue [87]. Many studies have documented the bio-accumulation of pesticides in fruits and vegetables, which allows the chemicals to enter the food chain and seriously harm both human and animal health [88,89,90,91]. Additionally, since soil and water bodies are closely connected, both surface water and groundwater are significantly affected by pesticide contamination. As a result, detectable pesticide levels have been identified mostly in groundwater and surface water streams in areas of agriculture and urban land activities [92]. Figure 5 shows some of the most commonly found pesticides in surface water.

Pesticides can have both short-term (acute) and long-term (chronic) effects on humans. Eye stinging, rashes, blindness, dizziness, nausea, diarrhea, and even death are some possible effects of acute toxicity, while different types of cancer, birth deformities, immunotoxicity, neurological and developmental toxicity, reproductive harm, and endocrine system disturbance are a few examples of documented chronic consequences [93]. Thus, it is crucial to extract and determine pesticide trace levels in various environmental matrices using reliable and environmentally friendly methodologies. In this context, SBWE has shown to be an effective, rapid, and green strategy for the recovery of pesticide-contaminated soils [94,95]. In comparison to PAHs and PCBs, pesticides are more soluble in water; hence, most are extracted at moderate temperatures. Miller et al. investigated the effects of temperature and pressure on the solubility of pesticides in water and found that moderate pressure had little impact on their solubilities, but that every 50 °C increase in temperature increased the solubility of the pesticides by about one order of magnitude [96]. Table 3 shows the extraction of various pesticides from environmental matrices using SBWE.

An investigation was carried out by Jimenez-Carmona et al. to compare the effectiveness of SBWE and SFE in the extraction of trichloropyridinol, a metabolite of chlorpyrifos, from soil [97]. They achieved 95% extraction by the SFE method at 40 °C and 383 bar in 30 min, utilizing organic additives. Nevertheless, complete extraction was possible with SBWE at 250 °C and 200 bar in just 15 min, negating the need for any further additives. Subsequently, in a more thorough investigation, Luque-Garcial et al. and Crescenzi et al. used the SBWE approach at a low temperature to extract a wide number of pesticides from the soil with outstanding recoveries [98,101]. Crescenzi et al. extracted 16 out of the 18 herbicides at 90 °C from the soil, which exhibited recoveries that varied from 81 to 93% [98]. At 85 °C, Luque-Garcia et al. extracted bentazone, 2,4-dichlorophenoxyacetic acid (2,4-D), 3,5,6-trichloro-2-pyridinyloxyacetic acid (triclopyr), 2,4,5-trichlorophenoxyacetic acid (2,4,5-T), and 2(2,4,5-trichlorophenoxy) propionic acid (2,4,5-TP) with recoveries of 94.2–113.1% at 60 min [104]. Almost identical to these data, Konda et al. indicated that at 105 °C, pesticide recoveries varied between 84.6 and 91.1% (acetochlor, atrazine, carbendazim, imidacloprid, and isoproturon), except for diazinon, which was recovered at 59.4% from soil [106]. Corcia et al., on the other hand, observed substantially greater recoveries, with extraction recoveries for terbuthylazine (CBET) and its degradation products (DPs) from an aged soil at 100 °C ranging between 95 and 103% [107]. Furthermore, as compared to Soxhlet extraction and double extraction techniques, the extraction volumes achieved using SBWE were much higher. In a pilot-scale subcritical water extraction of pesticide-contaminated soil (8 kg), Lagadec et al. efficiently removed all pesticides (>99%), reducing their initial concentration of 400 ppm to below detectable levels (0.1 ppm) in 15 min at 250 °C (100 bar) [48]. Lou et al. determined chlorinated acid herbicides and their esters in sea sand and agricultural soil using static SBWE paired with a strong anion exchange (SAX) disk [99]. Quantitative recoveries, usually over 80%, were attained using static SBWE/SAX disk extraction at 100 °C for 30 min. Krieger et al. investigated the utilization of SBWE for extracting tricyclazole from soils and sediments [100]. At optimal conditions (150 °C and 30 min) using SBWE, the extraction recoveries of tricyclazole from soil and sediment were 85–100%, regardless of the incubation time and sample matrix, except for one sediment. Mcgowin et al. determined that an extraction time of 20 min and a temperature of 110 °C provided the maximum extraction recovery ranging from 74 to 91% when they utilized static SBWE and SPE to evaluate pesticides in certified reference sediment (CRM 104) [53].

Extracting less-polar pesticides via SBWE requires higher temperatures; Eskilsson et al. revealed that at 100 °C, the recoveries for carbofuran, imidacloprid, and carbosulfan were 81, 98, and <1%, respectively [102]. However, SBWE may extract polar molecules more successfully in comparison to the large range of organic solvents. With recoveries of 77% at 150 °C using SBWE compared to traditional organic extraction (69%) and supercritical fluid extraction (45%) at 140 °C, Krieger et al. observed superior extraction recoveries for cloransulam-methyl from Wayside, MS (1DAT) soils [108].

To alter the effectiveness of some pesticides’ extraction, organic solvents (acetone, methanol, acetonitrile) could be added to the water in SBWE. Thus, using water-acetonitrile as the extraction solvent, Rodil et al. observed 4.1–85.2% recoveries for organochlorine pesticides and chlorobenzene from spiked soil samples (25–155 ng/g) at 120 °C in pressurized SBWE paired with stir bar sorptive extraction (SBSE) [109]. Similarly, Chienthavorn et al. conducted a comparative study using pure water and a modifier in the SBWE method to extract insecticides, herbicides, and fungicides from soil, sediment, and sand samples [103]. The optimal extraction temperature was between 120 and 180 °C, at which point the recoveries from sand samples ranged from 96% to 101% for most pesticides, except for butachlor. The recoveries were over 91% from sediment samples using an organic modifier. The extraction method was also used on soil samples, where the majority of the pesticides recovered between 82% and 105%, except dieldrin, which recovered 76%.

In 2013, Islam et al. reported an extraction efficiency of 99.9% of pesticides (diazinon, parathion, phenthoate, and EPN) at 150 °C and 2 MPa in just 20 min from contaminated soil [104]. Then, in 2017, Zhao et al. developed a green and selective extraction method for triazine herbicides based on a combination of SBWE and molecularly imprinted solid phase extraction (MISPE) [105]. Liquid chromatography–tandem mass spectrometry (LC-MS/MS) was employed to analyze herbicides. It was discovered that 15% ethanol as the organic modifier and 150 °C for 15 min were the optimum extraction conditions for triazine herbicides. In addition, molecularly imprinted polymers (MIPs) were added during SBWE, which increased the extraction efficiency. Therefore, compared to employing SBWE-MISPE alone (30% to 67%), adopting the optimized MIP/SBWE-MISPE approach exhibited superior recoveries (78.9% to 101%).

### 2.4. Other OPs

Pharmaceuticals are primarily used to treat, prevent, and diagnose diseases in humans and animals. However, over time, pharmaceutical production and consumption have grown, making them one of the emerging environmental contaminants [110,111]. Due to their widespread detection, their ongoing release into ecosystems has grown into a severe problem, causing serious health effects in humans, animals, and plants. They have been detected in drinking water [112], surface water [113], groundwater [114], marine waters [115], and soils [116]. They have also been shown to bio-accumulate in fruits and vegetables [117,118]. Pharmaceuticals can be removed from water using a variety of traditional procedures, including physical, chemical, and biological treatments [119]. However, the majority of them are unable to remediate pharmaceuticals due to the low concentration of pharmaceuticals in water, which has resulted in the development of new and sophisticated treatment methods. Lately, there has been significant interest in using polymer-based technology to remove drugs from the environment [120]. To extract and eliminate pharmaceuticals from environmental matrices, several extraction procedures have also been developed. There have been comparatively fewer investigations of SBWE of pharmaceuticals from the environment compared to various other methods. However, SBWE is an environmentally friendly technique that has shown promise in extracting and removing pharmaceuticals from environmental matrices with the highest removal efficiency. Richter et al. reported employing the SBWE method to extract nifedipine from a synthetic pharmaceutical formulation, with a 99.2% recovery rate achieved in under 20 min at 150 °C [121]. Yabalak et al. studied the degradation of 6-aminopenicillanic acid and cloxacillin in aqueous solution by SBW and oxidizing agents such as O_2_, H_2_O_2_, and K_2_S_2_O_8_ [122]. The highest TOC removal rates for 6-aminopenicillanic acid (83.54%) and cloxacillin (76.02%) utilizing H_2_O_2_ and K_2_S_2_O_8_, respectively, were reported. Similarly, Emire et al. demonstrated that with the SBW approach and H_2_O_2_ as an oxidant, paracetamol was 100% degraded [123].

Phthalates esters (phthalates) a predominant type of plasticizer, are a common class of environmental contaminants that are added to polyvinyl chloride (PVC) to give it more flexibility and hardness [124,125]. Each year, more than 18 billion pounds of phthalates are used in products related to medicine and lab work, construction materials, printing inks, cosmetics and personal hygiene, clothing, food items, and packaging, among other things [126,127,128,129,130]. Phthalate pollution exposure has extremely detrimental impacts on human health, one of which is the disturbance of hormone levels during fetal development [131,132]. Given their hazardous nature, phthalates have been extracted from environmental samples using a variety of analytical techniques, including SBWE. In a study, Chang et al. investigated the extraction of different types of phthalates from soil samples using SBWE [133]. The removal efficiency of phthalates was obtained at 80–90% under the optimum conditions of 250 °C and 10 MPa. According to Colnik’s report on the degradation of polyethylene terephthalate wastes using SBW, the maximum yield of terephthalic acid was found to be 85–90% at 300 °C and a 30 min reaction duration [134]. Similarly, at 300 °C, Xiu et al. achieved 99.2% decomposition of diethylhexyl phthalate-rich PVC waste at just 15 min using SBW [135].

Crude oils and refined petroleum products are a very complex mixture of different types of organic compounds, including aliphatic, aromatic, and polar compounds containing nitrogen, oxygen, and sulfur [136]. Due to the massive amounts of crude oil released by oil mining, the refining industry, transportation, and utilization, crude oil is one of the principal environmental pollutants that harm the aquatic and terrestrial environments [137,138,139]. SBWE is a sophisticated analytical technique that could potentially be applied to crude oil pollution investigations and remediation. The application of SBWE to the remediation of lubricating oil from contaminated soil by Islam et al. resulted in an exceptional removal efficiency of 99% at 275 °C in 150 min [140]. In another study, Islam et al. reported 99.8% extraction efficiency of diesel from crude oil-contaminated soil in 60 min at 200 °C using SBWE [141]. According to Taki et al., 99.69% and 87.33% of the crude oil was recovered and removed, respectively, from contaminated soil at 250 °C and 120 min [139].

### 2.5. Comparison of SBWE with Other Conventional Techniques

Over the years, a number of conventional techniques have been developed to extract different types of contaminants from environments. Pesticides, PAHs, PCBs, pharmaceuticals, and others are extracted from the environment using a variety of techniques, such as microwave extraction, ultrasonic extraction, and Soxhlet extraction [142,143,144]. However, low extraction efficiency, large organic solvent consumption, low reproducibility, extended extraction duration, and solvent diffusion are some of their drawbacks. Nevertheless, SBWE is a potentially effective green method that uses commonly available, harmless water at critical pressures and temperatures to extract organic contaminants from environmental matrices. This technique has several other advantages, such as high extraction efficiency, rapid extraction, low process cost, and effectiveness on both polar and non-polar OPs. For example, SBWE extracted PAHs with up to 99% recoveries in just 60 min [51], whereas Soxhlet extraction using an organic solvent produced recoveries that were comparable in 16 h [145]. The pesticide extraction methods of microwave, Soxhlet, and SBWE showed nearly comparable recoveries [105,146]. However, the extraction times for Soxhlet and SBWE were 900 min and 20 min, respectively. Table 4 contrasts the application of SBWE with other conventional methods including microwave, Soxhlet, and ultrasound for the extraction of PAHs, PCBs, pesticides, and pharmaceuticals from environmental matrices.

## 3. Conclusions

SBWE is a promising green technique for the extraction of organic pollutants from environmental matrices using nontoxic and widely available water at critical temperatures and pressures. The primary benefits of SBWE are attributed to the usage of water due to its tunable polarity in a critical state instead of other toxic organic solvents. Thus, by adjusting extraction conditions (pressure and temperature), a variety of polar or less-polar OPs, including PAHs, PCBs, and pesticides, can be efficiently removed from different environmental matrices (soils, sand, sediments, and water) within a short time with excellent recoveries. The effectiveness of this approach to remove pesticides, PAHs, and PCBs is not significantly affected by pressure. However, temperature variations have a significant impact on the extraction effectiveness of these compounds in the SBWE process, depending on their polarity. Pesticides, being more soluble in water, are typically extracted at moderate temperatures, with the optimal condition falling between 90 and 150 °C. Conversely, PAHs and PCBs, which exhibit poor water solubility, require higher temperatures for optimal extraction. Lower molecular weight PAHs show optimum extraction between 150 and 200 °C, while higher molecular weight PAHs necessitate temperatures between 200 and 300 °C. For PCBs, solubility decreases with increased chlorination, and the optimal range for extraction lies between 250 and 300 °C. Occasionally, organic modifiers such as methanol, ethanol, acetone, and acetonitrile are introduced to enhance the efficiency of SBWE extraction and maximize the recovery of OPs from complex environmental matrices.

## 4. Future Developments

Subcritical water has demonstrated great potential to replace toxic organic solvents in chemical extractions, chromatography, synthesis, environmental remediation, and other chemical processes. To further develop subcritical water technology, more fundamental research, such as the determination and prediction of organic solubility in subcritical water, as well as organic decomposition under subcritical water conditions, are required.

It has to be pointed out that the solubility of nonpolar organics in water is still poor at mild subcritical temperatures, and thus, organic modifiers may be needed to achieve efficient subcritical water extraction. However, toxic organic modifiers such as methanol or acetonitrile must be avoided. Good alternatives are ethanol or acetone since they are much less toxic.

## Figures and Tables

**Table 1:** SBWE of PAHs from environmental matrices.

Environmental Matrices Type	Type of Extracted PAHs	Extraction Conditions			% Recovery (% RSD)	Removal (%)	Ref.
		Temp (°C)	P (bar)	Time (min)			
Spiked Sand	Naphthalene	250	50	15	>90%	100	[14]
	Phenanthrene					96	
	Anthracene					95	
	Chrysene					90	
	Benzo[a]pyrene					86	
	Benzo[ghi]perylene					94	
Railroad Bed Soil	Naphthalene	250	-	60	278 (7)	-	[52]
	Acenaphthene				210 (6)		
	Phenanthrene				99 (12)		
	Anthracene				324 (14)		
	Fluoranthene				91 (10)		
	Pyrene				104 (10)		
	Benz[a]anthracene				153 (13)		
	Chrysene				100 (9)		
	Benzo[a]pyrene				98 (22)		
	Perylene				161 (11)		
	Benzo[ghi]perylene				80 (16)		
Petroleum Waste Sludge	Naphthalene	250	50.7	60	126 (10)	49	[23]
	Phenanthrene				109 (15)	77	
	Pyrene				82 (31)	87	
Soil	Naphthalene	275	100	60	-	>99	[48]
	Acenaphthene						
	Chrysene						
	Benz[a]anthracene						
	Benzo[b+k]fluoranthene						
	Benzo[e]pyrene						
	Benzo[a]pyrene						
	Indeno[1,2,3-cd]pyrene						
	Benzo[ghi]perylene						
Soil	Naphthalene	250	-	60	96	>90%	[51]
	2-Methyl naphthalene				99		
	1-Methyl naphthalene				99		
	Acenaphthene				93		
	Phenanthrene				94		
	Anthracene				93		
	Fluoranthene				99		
	Pyrene				91		
	Benzo[a]anthracene				94		
	Chrysene				99		
	Benzo[b+j+k]fluoranthene				93		
	Benzo[a]pyrene				93		
	Perylene				93		
	Indeno[1,2,3-cd]pyrene				92		
	Benzo[ghi]perylene				92		
Soil	Pyrene	150	50	15 + 10	101.8 (10.4)	-	[53]
	Benzo[a]anthracene				73.6 (11.5)		
	Benzo[e]acenaphthen				96.8 (10.2)		
	Benzo[k]fluoranthene				110.4 (7.4)		
	Benzo[a]pyrene				106.5 (9.3)		
	Benzo[ghi]perylene				104.0 (1.2)		
Sediment (CRM 104)	Benz[a]anthracene	150	-	20	88 (5)	-	[54]
	Benzo[b]fluoranthene				55 (4)		
	Benzo[k]fluoranthene				104 (4)		
	Benzo[ghi]perylene				95 (1)		
	Benzo[a]pyrene				89 (3)		
	Chrysene				87 (4)		
	Fluoranthene				89 (12)		
	Indole[1,2,3-cd]pyrene				106 (1)		
	Phenanthrene				87 (6)		
	Pyrene				101 (12)		
Soil	Naphthalene	150	100	30	-	99.61	[55]
	Phenanthrene	300				98.12	
	Fluoranthene					96.24	
	Pyrene					94.05	
Soil	Naphthalene	200	-	60	-	100	[56]
	Phenanthrene	250				96	
	Fluoranthene					96	
	Pyrene					98	
Soil	Phenanthrene	275	40	60	-	99	[57]
	Fluoranthene					92	
	Pyrene					91	
Soil	Phenanthrene	165	20	15	-	83.58	[58]
Soil	Benzo[a]pyrene	250	101.3	30	-	96	[59]

P, pressure; Temp, temperature; RSD, relative standard deviation; Ref, reference.

**Table 2:** SBWE of PCBs from environmental matrices.

Environmental Matrices Type	Type of Extracted PCBs	Extraction Conditions			Removal (%)	% Recovery (%RSD)	Ref.
		Temp (°C)	P (bar)	Time (min)			
Sediment	PCB-26	250	50.7	15	>99	74 (11)	[73]
	PCB-28					106 (13)	
	PCB-44					101 (3)	
	PCB-52					100 (15)	
	PCB-102					88 (9)	
	PCB-118					89 (23)	
	PCB-149					ND	
	PCB-153					ND	
	PCB-105					ND	
	PCB-128					73 (18)	
	PCB-156					ND	
	PCB-180					73 (25)	
Industrial Soil	PCB-28	250	50.7	15	>99	95 (15)	[73]
	PCB-52				>99	91 (11)	
	PCB-101				96	92 (9)	
	PCB-118				92	84 (10)	
	PCB-149				92	79 (5)	
	PCB-153				85	81 (9)	
	PCB-105				94	91 (8)	
	PCB-138				88	74 (9)	
	PCB-128				91	70 (7)	
	PCB-156				82	71 (16)	
	PCB-180				73	70 (19)	
	PCB-170				71	71 (18)	
Sea Sand	PCB-101	250	253.3	-	-	87.5 (6)	[74]
	PCB-138					87.9 (5)	
	PCB-180					87.7 (2)	
	PCB-194					90.0 (3)	
Sand	PCB-2	250	-	-	-	102 (12)	[75]
	PCB-29					90 (13)	
	PCB-52					100 (23)	
	PCB-101					95 (30)	
	PCB-153					87 (35)	
	PCB-180					90 (23)	
Soil	PCB-118	250	-	60	>99.5	-	[76]
	PCB-28						
	PCB-31						
	PCB-44						
	PCB-52						
	PCB-101						
	PCB-118						
	PCB-138						
	PCB-149						
	PCB-153						
	PCB-170						
	PCB-180						
	PCB-194						
	PCB-209						

P, pressure; Temp, temperature; RSD, relative standard deviation; Ref, reference; ND, not detected.

**Table 3:** SBWE of pesticides from environmental matrices.

Environmental Matrices Type	Type of Extracted Pesticides	Extraction Conditions			% Recovery (%RSD)	Removal (%)	Ref.
		Temp (°C)	P (bar)	Time (min)			
Soil	Trichloropyridinol	250	200	15	99.7	100%	[97]
Soil	Cynarine	90	-	-	87	-	[98]
	Simazine				89		
	Atrazine				89		
	Isoproturon				88		
	Diuron				86		
	Linuron				84		
	Clopyralid				93		
	Picloram				91		
	Dicamba				90		
	Bentazone				85		
	MCPA				87		
	2,4-D				85		
	Mecoprop				84		
	Diclorprop				86		
	Bromoxynil				84		
	Ioxynil				84		
	2,4-DB				63		
	MCPB				62		
Sea Sand	4-Nitrophenol	100	-	30	85 (1.5)	-	[99]
	Pentacholorphenol				92 (1.0)		
	Dinoseb				42 (12)		
	3,5-Dichlorobenzoic acid				93 (2.5)		
	Dicamba				92 (0.5)		
	2,4-DP				99 (0.4)		
	2,4-D				103 (1.2)		
	2,4,5-TP				100 (1.0)		
	2,4,5-T				101 (1.9)		
	2,4-DB				93 (3.9)		
	Chloramben				90 (2.2)		
	Picloram				69 (9.9)		
	Acifluorfen				108 (7.3)		
	2,4-Dichlorophenylacetic acid				92 (2.5)		
Agricultural Soil	4-Nitrophenol	100	-	30	90 (6.2)	-	[99]
	Pentacholorphenol				88 (7.6)		
	Dinoseb				47 (11)		
	3,5-Dichlorobenzoic acid				90 (6.1)		
	Dicamba				76 (6.8)		
	2,4-DP				96 (6.1)		
	2,4-D				93 (6.3)		
	2,4,5-TP				92 (6.3)		
	2,4,5-T				92 (7.6)		
	2,4-DB				90 (6.1)		
	Chloramben				90 (8.0)		
	Picloram				71 (7.6)		
	Acifluorfen				95 (9.5)		
	2,4-Dichlorophenylacetic acid				88 (4.9)		
Soil and Sediment	Tricyclazole	150	-	30	85–100	-	[100]
Soil	Trifluralin	250	-	15	-	>99	[48]
	Atrazine						
	Alachlor						
	Metolachlor						
	Cyanazine						
	Pendimethalin						
Sediment	Ametryne	110	-	20	78 (4)	-	[54]
	Atrazine				89 (12)		
	Carbaryl				74 (5)		
	Chlorpyrifos				91 (3)		
	Trifluralin				90 (3)		
Soil	Bentazone	85	-	60	94.2–113.1	-	[101]
	2,4-D						
	Triclopyr						
	2,4,5-T						
	2,4,5-Tp						
Dust waste	Carbofuran	100	25	30	81 (2.9)	-	[102]
	Imidacloprid				98 (1.0)		
	Carbosulfan				<1		
Soil	Chlordane	120	-	10	99 (13)	-	[103]
	Malathion	160			87 (12)		
	Heptachlor				82 (4)		
	Aldrin			20	89 (2)		
	Dieldrin				77 (7)		
	Butachlor			10	82 (11)		
	Metalaxyl				94 (6)		
	Propiconazole				104 (4)		
	Thiobencarb	180			82 (12)		
Soil	Diazinon	150	20	20	-	100	[104]
	Parathione					100	
	Phenthoate					100	
	EPN					99	
Soil	Ametryn	150	-	20	78.9–101	-	[105]
	Promtryn						
	Simetryn						
	Methoprotryn						
	Simazine						
	Atrazine						
	Propazine						
	Terbuthylazine						

P, pressure; Temp, temperature; RSD, relative standard deviation; Ref, reference.

**Table 4:** Comparison of SBWE with other conventional methods regarding the extraction of PAHs, PCBs, pesticides, and pharmaceuticals from the environment.

Analyzed Compounds	Extraction Method	Extraction Conditions				% Recovery (%RSD)	Ref.
		Temp (°C)	P (bar)	Time (min)	Solvent		
PAHs	SBWE	250	-	60	Water	92–99 (-)	[51]
	Microwave	-	-	6	Acetonitrile	78.7–115.6 (0.7–7.8)	[147]
	Soxhlet	-	-	960	DCM/acetone	57–99 (-)	[145]
	Ultrasound	-	-	45	n-Hexane	29.2–82.5 (6.5–47.1)	[148]
PCBs	SBWE	250	-	-	Water	87–102 (12–35)	[75]
	Microwave	115	-	10	Hexane/acetone	81–93 (-)	[149]
	Soxhlet	-	-	540	Methylene chloride	64.3–75.2 (-)	[150]
	Ultrasound	-	-	45	Methylene chloride	69.2–77.2 (-)	
Pesticides	SBWE	150	-	20	Water	78.9–101	[105]
	Microwave	110	-	10	Hexane/acetone	84.98–104.06 (0.52–9.30)	[146]
	Soxhlet	-	-	900	Hexane/acetone	86.79–105.12 (0.61–12.12)	
	Ultrasound	-	-	10	Acetonitrile	75–111 (-)	[151]
Pharmaceuticals	SBWE	150	-	20	Water	99.2 (1.9)	[121]
	Microwave	65	-	15	Methanol/water	40–100 (<5)	[152]
	Soxhlet	-	-	480	Acetonitrile	88–108 (-)	[153]
	Ultrasound	-	-	45	Ethylacetate/formic acid	>80 (1.1–10)	[154]

## Data Availability

Not applicable.
